# Cochlear implant artifact analysis in cranial MRI: effects of field strength and imaging sequence

**DOI:** 10.3389/fneur.2026.1861544

**Published:** 2026-07-17

**Authors:** Siqi Gao, Kaiyue Zhao, Longtao Ma, Jun Lin, Dongmei Wang, Jiayu Ni, Dian Yang, Jiaying Wang, Zili Yin, Mengxue Zhang, Yiqi Ma, Hongjian He, Yuan Li

**Affiliations:** 1 Department of Otorhinolaryngology, Affiliated Hospital of Hangzhou Normal University, Hangzhou, Zhejiang, China; 2 Hangzhou Normal University, Hangzhou, Zhejiang, China; 3 Department of Radiology, Affiliated Hospital of Hangzhou Normal University, Hangzhou, Zhejiang, China; 4 College of Biomedical Engineering and Instrument Science, Zhejiang University, Hangzhou, Zhejiang, China; 5 Zhejiang Nurotron Biotechnology Co., Ltd., Hangzhou, Zhejiang, China; 6 Central Hospital, Jinhua, Zhejiang, China; 7 School of Physics, Zhejiang University, Hangzhou, Zhejiang, China; 8 Yiwu The Center for Caribbean Studies at Hangzhou Normal University, Hangzhou, Zhejiang, China

**Keywords:** artifact, cochlear implant, field strength, image quality, imaging sequence, magnetic resonance

## Abstract

**Objective:**

To investigate the artifacts produced by the cochlear implant (CI) CS-30A in nonimplanted human clinical trials under different magnetic resonance (MR) field strengths and imaging sequences.

**Methods:**

The CI was placed at a standardized surface projection location on the temporal region of 12 healthy subjects. Axial cranial MR scans were performed at both 1.5 T and 3.0 T field strengths using the following sequences: T1-weighted spin-echo (SE), T2-weighted fast spin-echo (FSE), and T2*-weighted gradient-echo (GRE). The maximum dimension of CI artifacts was measured, and the artifact area was quantified using both manual measurement and a deep learning–based automatic segmentation algorithm. The impact of CI artifacts on the visibility of brain anatomy was evaluated by radiologists.

**Results:**

Under both 1.5 T and 3.0 T MR scanning, GRE sequences exhibited significantly larger artifact maximum dimension and area than SE and FSE sequences (*p* < 0.05). No significant differences were noted in maximum artifact dimensions between 1.5 T and 3.0 T MR across sequences (*p* > 0.05). The difference in artifact area between manual measurement and the deep learning–based automatic segmentation algorithm was not significant on SE sequences (*p* = 0.377).

**Conclusion:**

GRE sequences produced a wider range of CI artifacts than SE and FSE sequences at both 1.5 T and 3.0 T. No significant difference in artifact size was observed between the two field strengths. The artifact areas on SE sequences showed high consistency between manual measurement and the deep learning–based automatic segmentation algorithm. Therefore, SE or FSE sequences should be prioritized over GRE for CI recipients, and both 1.5 T and 3.0 T are clinically feasible.

## 1 Introduction

Magnetic resonance imaging (MRI) has become the primary first-line diagnostic modality for assessing various brain disorders and neurological pathologies owing to its distinct advantages, including the absence of ionizing radiation, superior soft tissue contrast resolution, and high sensitivity to numerous pathological conditions (1).

The cochlear implant (CI), a neuroprosthetic device designed to provide auditory input for individuals with hearing impairment, offers considerable benefits for patients with severe to profound sensorineural hearing loss of cochlear origin. Currently, approximately 1 million people have received CIs worldwide (2). Furthermore, with the expanding indications for CI implantation and the increasing need for postoperative evaluation of intracranial conditions such as vestibular schwannomas, CI recipients have an estimated 50–75% probability of requiring an MRI over their lifetime (3).

Nonetheless, the presence of the CI has also raised concerns about MRI safety. Historically, MRI has been viewed as an absolute contraindication for CI recipients (4). The interaction between the internal magnet of the CI and the magnetic field of MRI can lead to several adverse events, including magnet demagnetization or displacement, polarity reversal, patient discomfort or pain, image artifacts, and even potential device malfunction (5–10). Certain solutions have been proposed in this regard, such as tightly wrapping the head of CI recipients and creating a new generation of CI models with magnetic systems that can withstand magnetic fields, allowing CI recipients to undergo MRI scans (11–13). However, the clinical imaging of the head remains disturbed by a large number of artifacts (11, 14, 15). Similar MRI artifact challenges have been reported for other implantable hearing devices, such as bone conduction hearing aids (16, 17).

NUROTRON (Zhejiang, China), a CI manufacturer, recently developed the CS-30A implant (18). Rotatable magnet designs, which allow the internal magnet to align with the external magnetic field and reduce torque and displacement forces, have become common practice across major cochlear implant manufacturers (12, 13, 19). The magnet is enclosed within a non-magnetic housing, allowing it to freely rotate and automatically align with the external magnetic field during scanning. This design effectively reduces torque and displacement forces, thereby minimizing patient discomfort and the risk of magnet dislocation. In addition, the rotatable configuration helps decrease the extent of susceptibility artifacts, making MRI evaluation possible without magnet removal. This study aimed to determine the artifacts of CS-30A in nonimplanted human clinical trials under different magnetic field strengths and MRI sequences, providing supplementary evidence to select the optimal MRI protocol.

## 2 Materials and methods

### 2.1 Participants

The study protocol was conducted in strict adherence to the Declaration of Helsinki and it was approved by the Ethics Committee of the Affiliated Hospital of Hangzhou Normal University (Approval No.: 2024(E2)-KS-153). All participants provided their signed informed consent. No adverse events either potentially or directly related to the study or the test device occurred during the trial, and no participants withdrew prematurely. A total of 12 healthy volunteers participated in the study, of whom 6 were males and 6 females of age 23–27 years (mean age: 25.05 ± 1.19 years). The soft tissue thickness at the implant fixation site was retrospectively measured on axial T1-weighted SE images at the location 9 cm posterior to the external auditory canal on the right temporal side, corresponding to the CI magnet placement site. The mean thickness was 7.3 ± 1.2 mm (range: 5.0–8.9 mm). Head circumference was measured using a non-elastic tape measure placed at the level of the glabella and the external occipital protuberance, following standard clinical practice. The mean head circumference of the participants was 56.4 ± 1.5 cm (range: 54.2–59.0 cm).

The patient inclusion criteria include the following: (1) age 18–60 years; (2) no underlying medical conditions; (3) willingness to participate in the trial and provision of signed informed consent.

The exclusion criteria for the study were as follows: (1) allergy to implant materials; (2) patients with cardiac pacemakers, neurostimulators, metallic heart valves, or other electronically activated/magnetic field-sensitive implants; (3) presence of aneurysm clips (except for non-ferromagnetic materials such as titanium); (4) presence of metallic foreign bodies, including intraocular metal fragments, cochlear implants, metallic prostheses, artificial joints, or any ferromagnetic implants.

### 2.2 Equipment and imaging parameters

All participants underwent MRI scanning using both the 1.5 T SIEMENS Area scanner at Hangzhou Normal University Affiliated Hospital and the 3.0 T GE Discovery MR-750 scanner (GE Medical Systems, Waukesha, WI) at the Center for Cognition and Brain Disorders, Hangzhou Normal University.

All participants underwent MRI at both 1.5 T and 3.0 T using three sequences: T1-weighted spin-echo (SE), T2-weighted fast spin-echo (FSE), and T2*-weighted gradient-echo (GRE). The acquisition parameters for each field strength are summarized in Table 1.

**Table 1:** MRI acquisition parameters for each sequence at 1.5 T and 3.0 T.

Field strength	Sequence	TR	TE	FOV	THK	SP	BW	ETL	Matrix
1.5 T	T1 SE	500	14.0	24*24	6.0	1.5	121	—	512*256
1.5 T	T2 FSE	4,000	115.4	24*18	6.0	1.5	130	15	384*192
1.5 T	T2* GRE	420	20.0	24*18	6.0	1.5	130	—	256*192
3.0 T	T1 SE	500	14.0	24*24	3.0	1.5	121	—	512*256
3.0 T	T2 FSE	4,000	115.4	24*18	3.0	1.5	130	15	384*192
3.0 T	T2* GRE	420	20.0	24*18	3.0	1.5	130	—	256*192

TR, repetition time (ms); TE, echo time (ms); FOV, field of view (cm^2^); THK, slice thickness (mm); SP, slice gap (mm); NS, number of slices; BW, bandwidth (kHz/px); ETL, echo train length; matrix, matrix size. SE, spin-echo; FSE, fast spin-echo; GRE, gradient-echo. “*” indicates multiplication in the matrix size (e.g., 512×256 means 512 by 256 pixels).

All sequences were performed within the manufacturer’s MR safety guidelines for the Nurotron CS-30A implant (whole-body average SAR ≤ 2.0 W/kg at 1.5 T and 3.0 T under normal operating mode).

The SE and FSE parameters were based on our routine cranial MRI protocols. The GRE parameters followed standard GRE protocols for metal artifact assessment, with FOV and slice thickness matched to the SE and FSE sequences where feasible to allow fair comparison (20).

### 2.3 Experimental procedure

**Figure 1:**
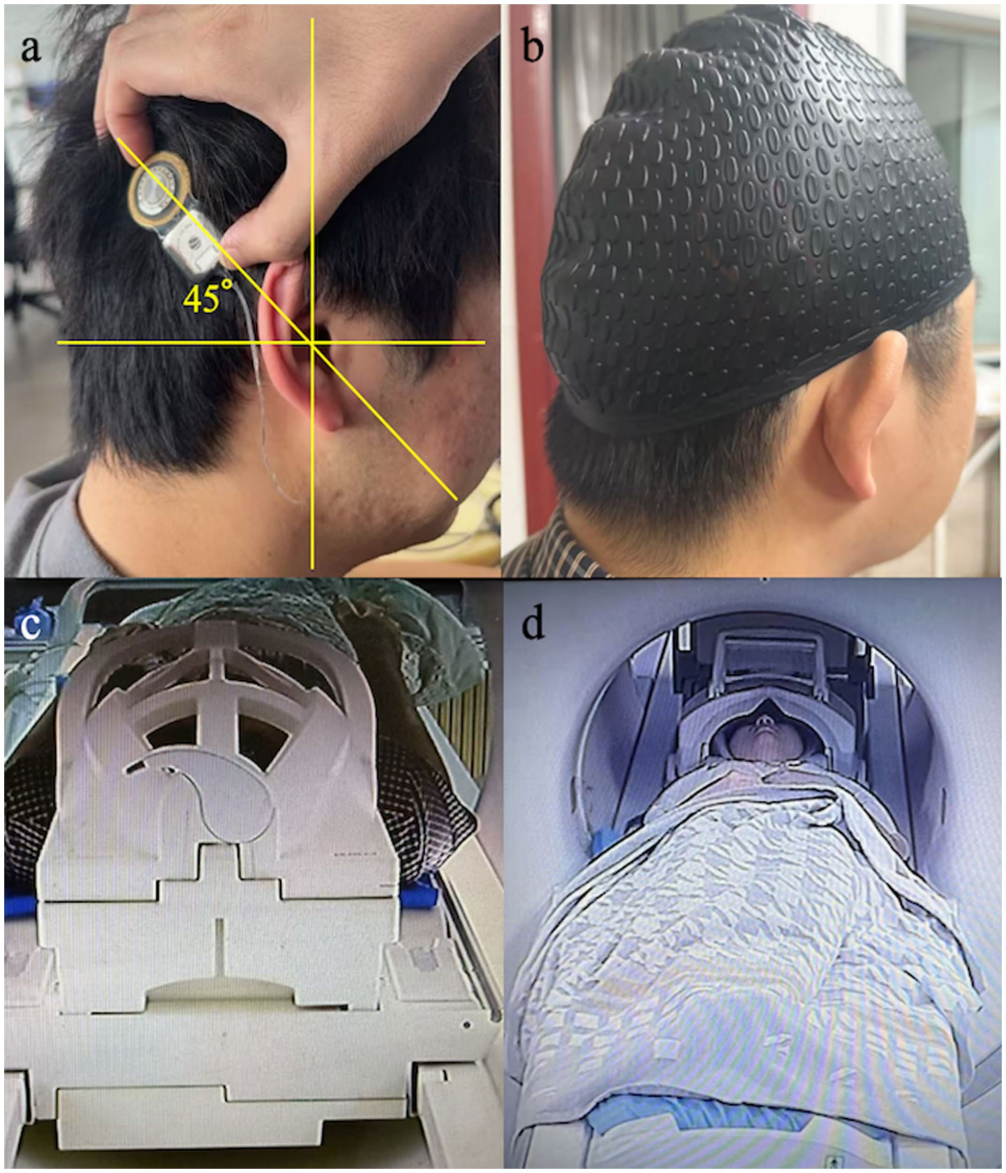
**(a,b)** The cochlear implant was fixed to the right temporal region of the head using a swim cap, and the implant position was marked on the surface of the cap **(c,d)** MRI scans were performed under 1.5 T and 3.0 T static magnetic field conditions.

The standard CI placement was simulated according to clinical practice. A horizontal line was drawn from the external auditory canal opening, and a vertical line was extended along the retroauricular horizontal groove. Subsequently, a 45° line was drawn from the intersection of the previous two lines, extending posteriorly and superiorly, to represent the orientation of the receiver–stimulator unit. The center of the internal magnet was placed approximately 9 cm posterior to the external auditory canal. The CS-30A implant was then fixed externally at the designated position on the right temporal region of each volunteer’s head using a swim cap (Figure 1a). This external fixation approach aligns with the methodology described by Dewey et al. (19). The placement location was marked by an experienced otolaryngologist using a surgical marker (Figure 1b). Each participant checked and adjusted the cap to ensure a secure fit and wore noise-reducing earplugs to minimize acoustic interference. The alignment of the implant with the skin marker was visually inspected before and after each MRI scan to confirm that no dislocation occurred. The participants were instructed to keep their eyes closed during the scanning procedure, remain motionless, and stay relaxed and awake. Each participant underwent MRI scanning under both 1.5 T and 3.0 T static magnetic field conditions (Figures 1c,d). Volunteers were queried about any sensations around the implant site, including discomfort, heat, vibration, or other implant-associated perceptions. This procedure was repeated for the remaining 11 participants.

### 2.4 Artifact measurement

The DICOM data of head MRI sequences from the 12 subjects were retrieved from the Picture Archiving and Communication System (PACS) and transferred to a Windows computer equipped with the MiniViewer software, which includes tools for magnetic resonance (MR) image viewing and distance measurement. These tools were used to measure the MRI artifacts created by the fixed CI in each subject, enabling comparisons between different series.

**Figure 2:**
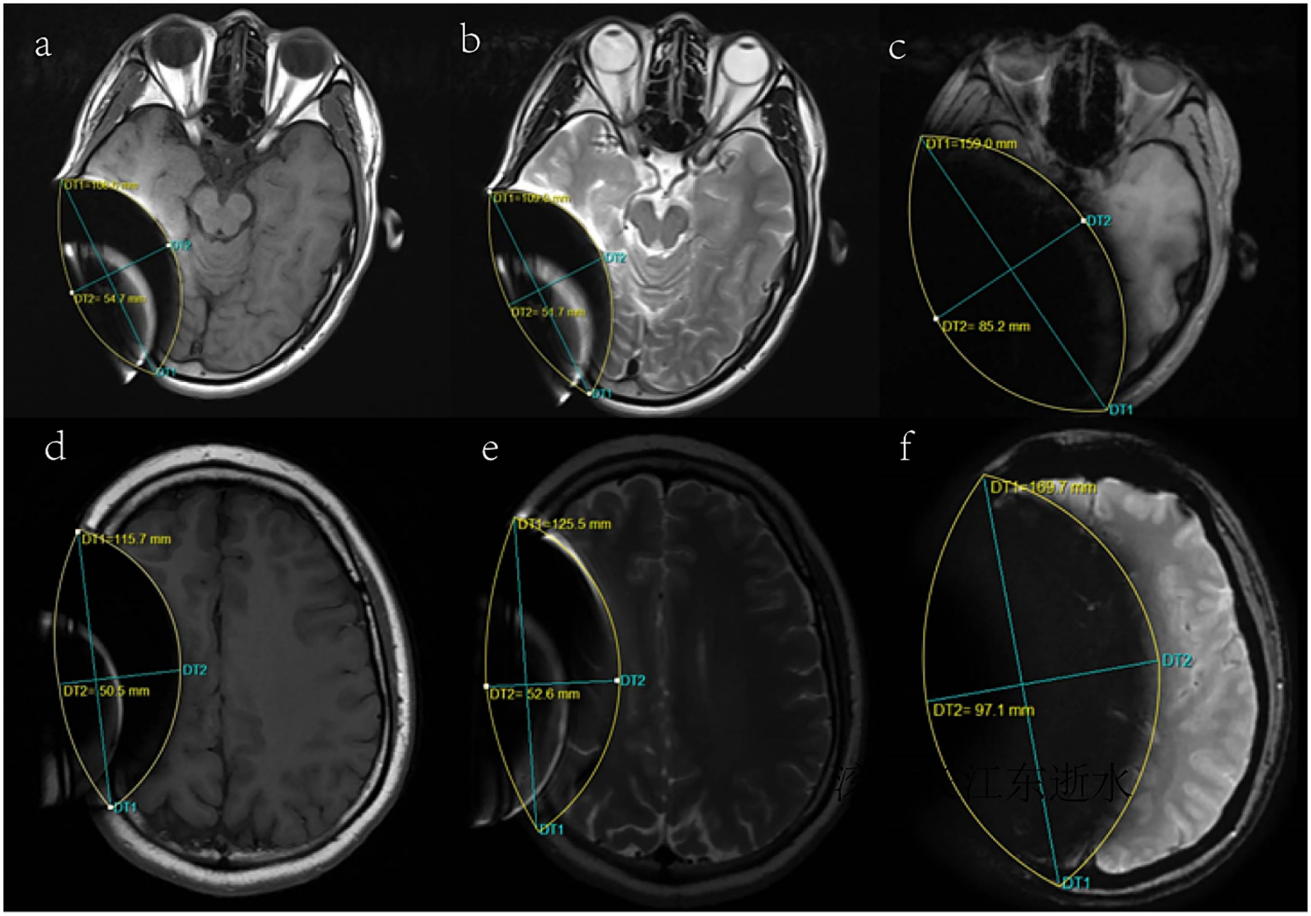
MRI artifacts across sequences in Volunteer 1 at 1.5 T and 3.0 T. **(a)** Signal void artifact on 1.5 T MRI (2D axial T1-weighted SE). **(b)** Signal void artifact on 1.5 T MRI (2D axial T2-weighted FSE). **(c)** Signal void artifact on 1.5 T MRI (2D axial T2-weighted GRE). **(d)** Signal void artifact on 3.0 T MRI (2D axial T1-weighted SE). **(e)** Signal void artifact on 3.0 T MRI (2D axial T2-weighted FSE). **(f)** Signal void artifact on 3.0 T MRI (2D axial T2-weighted GRE). The dashed lines indicate the two orthogonal measurements of the artifact (major and minor axes).

In the MiniViewer, a new window was opened for each imaging sequence of every subject. The slice with the most prominent artifact was selected on the axial images, and the artifact contour was carefully refined along the cerebral edge. Due to the irregular and often oblique shape of the artifact, the maximum artifact dimension was first identified regardless of orientation. A line was drawn along this maximum length. A second perpendicular line was then drawn at the midpoint of the first line to measure the orthogonal dimension (Figure 2). These two perpendicular measurements represent the major and minor axes of the artifact and were used for comparison across sequences, approximating the anteroposterior and mediolateral extents. The results of the maximum artifact dimension were recorded.

This measurement approach follows the core principles of the ASTM F2119 standard (21) for evaluating MRI artifacts from passive implants, specifically in measuring the maximum linear dimensions of the artifact.

To assess test–retest reliability, the same rater re-measured all artifact dimensions after a one-month interval. The intraclass correlation coefficient (ICC) was 0.976 (95% CI: 0.965–0.984, *p* < 0.001), indicating excellent test–retest reliability. The reported values are from the first measurement session.

### 2.5 Artifact area measurement

The DICOM data of MRI sequences from the 12 participants were retrieved from the PACS and transferred to a Windows computer for artifact area measurement using two approaches: (1) Manual measurement was performed in ImageJ by opening the slice with the most severe artifact, calibrating the scale using the embedded scale bar, manually outlining the artifact boundary with the polygon tool, and calculating the enclosed area. (2) An automated deep learning--based segmentation approach was used. The 12 subjects were randomly split at the subject level into 10 training and 2 validation sets. All sequences (SE, FSE, GRE) from the same subject were assigned exclusively to either the training or validation set to prevent data leakage. Manual annotation was performed by a radiologist according to ASTM F2119 standard (21) (signal intensity deviation >30% from normal tissue defined as artifact), with disagreements adjudicated by a senior neuroradiologist. A deep learning-based segmentation model (U-Net) was used to automatically outline the artifact boundary. The final artifact area was calculated by multiplying the number of pixels identified as artifact (N) by the pixel dimensions (Δx × Δy). To assess how well the automated segmentation agreed with manual annotation, we calculated several standard metrics: the Dice similarity coefficient (Dice), intersection over union (IoU), average symmetric surface distance (ASSD), Precision, Recall, and the 95th percentile Hausdorff distance (HD95).

### 2.6 Radiological evaluation of artifact impact on diagnostic utility

Two experienced neuroradiologists independently assessed the cranial MRI results of all 12 participants using a standardized four-point scoring system (0 = complete signal visibility with a high-quality view of the anatomical structures; 1 = > 50% signal visibility with partial artifact obscuration but still diagnostic; 2 = <50% signal visibility with partially discernible structures and nondiagnostic due to artifacts; 3 = complete invisibility), as previously described in cadaver studies on implantable hearing devices (16, 17). The following brain structures were evaluated: cerebellar hemispheres; pons/medulla; midbrain; deep gray matter (thalamus/basal ganglia); hippocampus; frontal, parietal, occipital, and temporal lobes; corpus callosum; internal auditory canal; and optic nerves. For cases showing discrepancy between the two primary raters, a third senior neuroradiologist was consulted as an arbitrator to provide the final definitive score.

The scoring scale ranged from 0 (completely visible high-quality anatomical view) to 3 (completely obscured). The final score for each structure, denoted as X, was determined as follows: when the two raters agreed, that score was used; when they disagreed, a third senior neuroradiologist provided the final score. The diagnostic usability of each structure was determined using the following thresholds: (1) X ≤ 0.75: high quality (HQ); (2) 0.75 < X ≤ 1.5: obscured by artifacts but still assessable for diagnostic purposes (A); (3) 1.5 < X ≤ 3: not assessable (NA). The ranges 1.5–2.25 and 2.25–3.0 were grouped, as both represented nondiagnostic image quality, whereas scores 0–1.5 were considered diagnostically usable.

The global mean quality scores across all MRI field strengths and sequence conditions were systematically calculated and visualized using Excel.

### 2.7 Statistical analysis

Statistical analysis was performed using SPSS version 27. Descriptive statistics are presented as mean ± SD. Normality was assessed using the Shapiro–Wilk test, and homogeneity of variances was tested using Levene’s test. The significance level was set at *α* = 0.05, with Bonferroni correction applied for multiple comparisons (α = 0.017 for three-group comparisons).

For continuous data (artifact dimensions and areas), parametric tests (paired t-test for two groups, repeated measures ANOVA for three groups) were used when data were normally distributed with equal variances; otherwise, non-parametric tests (Wilcoxon signed-rank test for two groups, Friedman test for three groups) were applied.

For the ordinal four-point scoring data (anatomical structure visibility scores), non-parametric tests were used: the Wilcoxon signed-rank test for field strength comparisons and the Friedman test followed by post-hoc Wilcoxon tests for sequence comparisons, with Bonferroni correction.

For comparisons between deep learning-based segmentation and manual measurements at 3.0 T, the paired t-test was used for normally distributed data with equal variances, and the Wilcoxon signed-rank test was used otherwise.

Effect sizes were reported as Cohen’s d with 95% CI for paired t-tests, and r = |Z|/√N for Wilcoxon tests.

In all analyses, *p* < 0.05 (or *p* < 0.017 after Bonferroni correction) was considered statistically significant.

## 3 Results

### 3.1 Artifact measurement

**Figure 3:**
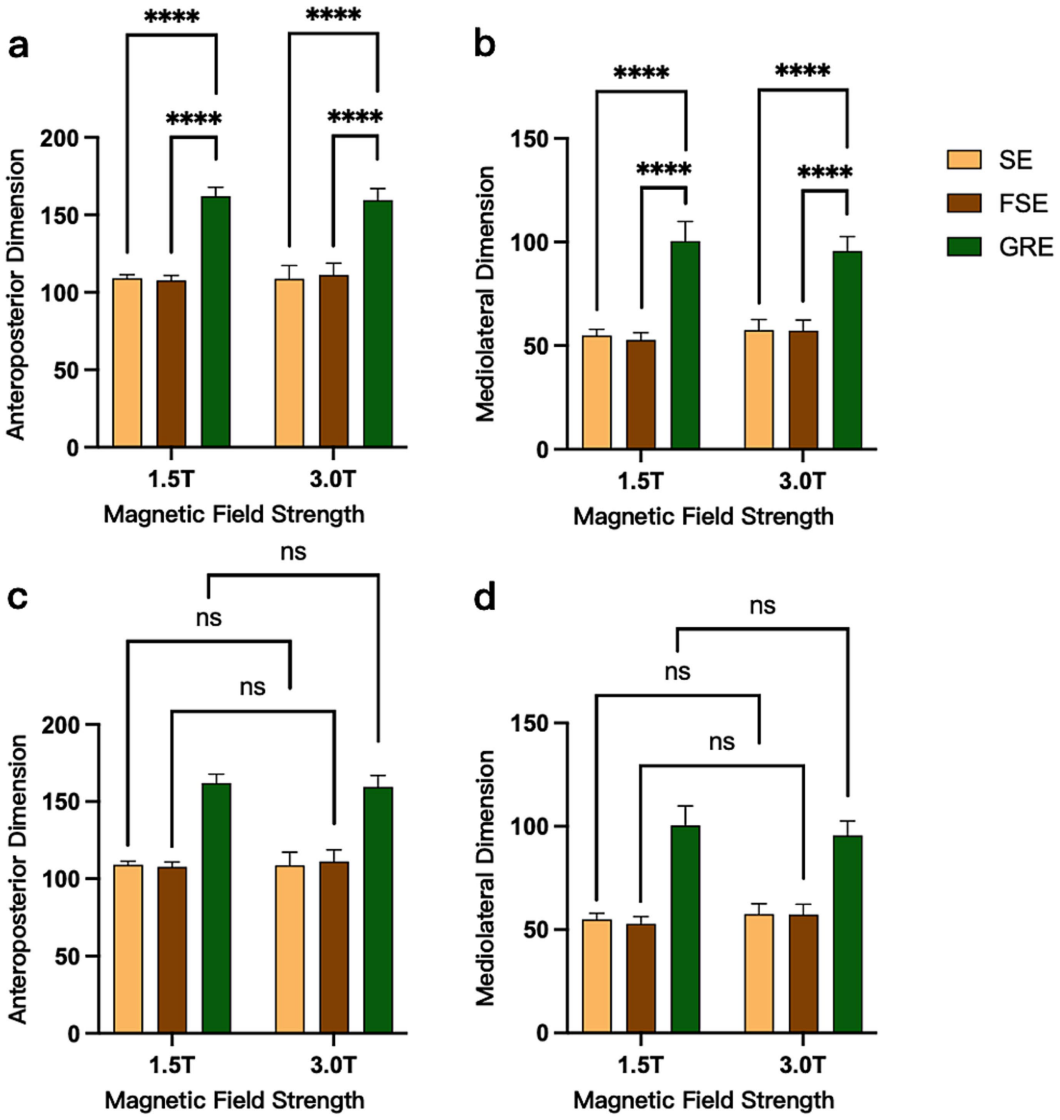
The maximum artifact dimension across MRI sequences at 1.5 T and 3.0 T. **(a)** Comparison of the maximum anteroposterior dimension between the sequences at 1.5 T and 3.0 T. **(b)** Comparison of the maximum mediolateral dimension between the sequences at 1.5 T and 3.0 T. **(c)** Comparison of the maximum anteroposterior dimension between 1.5 T and 3.0 T MR across sequences. **(d)** Comparison of the maximum mediolateral dimension between 1.5 T and 3.0 T MR across sequences ****p < 0.0001 “ns” means no significant difference.

The axial plane measurements at 1.5 T MRI demonstrated significant differences in the artifact dimensions across sequences: T1w SE display a maximum anteroposterior dimension of 109.02 mm (SD 2.32), T2w FSE 107.74 mm (SD 3.1), and T2w GRE 162.14 mm (SD 5.67) (*p* < 0.0001), whereas the maximum mediolateral dimensions were 54.93 mm (SD 3), 52.83 mm (SD 3.48), and 100.38 mm (SD 9.55), respectively (*p* < 0.0001). Similarly, at 3.0 T MRI, axial sequences exhibited significant variations: T1w SE anteroposterior dimension measured 108.73 mm (SD 8.6), T2w FSE 111.26 mm (SD 7.53), and T2w GRE 159.58 mm (SD 7.33) (*p* < 0.0001); with the corresponding mediolateral dimensions of 57.60 mm (SD 5), 57.31 mm (SD 4.98), and 95.67 mm (SD 6.83) (*p* < 0.0001). Shapiro–Wilk tests showed that all artifact dimension and area measurements were normally distributed (*p* > 0.05 for all). However, the sphericity assumption was violated at 1.5 T (p < 0.05), so the Friedman test was used. Post-hoc pairwise comparisons with Bonferroni correction (*α* = 0.017) are presented in Table 2. As shown in Figures 3a,b, GRE sequences consistently produced significantly larger artifacts than SE and FSE sequences across all conditions (all *p* < 0.01), while no significant differences were found between FSE and SE sequences.

**Table 2:** Pairwise comparisons of maximum artifact dimensions after Bonferroni correction (α = 0.017).

Field strength	Direction	Comparison	95% CI	*p* value	Effect size (r/Cohen’s d)
1.5 T	Anteroposterior	FSE vs. SE	—	0.182	r = 0.385
		GRE vs. SE	—	0.002	r = 0.884
		GRE vs. FSE	—	0.002	r = 0.883
	Mediolateral	FSE vs. SE	—	0.041	r = 0.591
		GRE vs. SE	—	0.002	r = 0.884
		GRE vs. FSE	—	0.002	r = 0.884
3.0 T	Anteroposterior	FSE vs. SE	[−7.83, 2.77]	0.315	d = −0.304
		GRE vs. SE	[−57.63, −44.09]	< 0.001	d = −4.775
		GRE vs. FSE	[−52.34, −44.31]	< 0.001	d = −7.643
	Mediolateral	FSE vs. SE	[−1.66, 2.25]	0.749	d = 0.095
		GRE vs. SE	[−44.11, −32.03]	< 0.001	d = −4.004
		GRE vs. FSE	[−43.83, −32.88]	< 0.001	d = −4.453

Bonferroni correction was applied (α = 0.017). For 3.0 T (paired t-tests), Cohen’s d and 95% CIs are reported.

No significant differences were noted in maximum artifact dimensions between 1.5 T and 3.0 T MR across sequences (*p* > 0.05) (Figures 3c,d).

### 3.2 Artifact area measurement

For axial plane acquisitions at 3.0 T MRI, the maximum artifact areas measured manually were 4543.15 mm^2^ (SD 647.03) for T1-weighted SE, 4579.95 mm^2^ (SD 535.30) for T2-weighted FSE, and 10463.94 mm^2^ (SD 1475.53) for T2-weighted GRE sequences. The corresponding measurements obtained through the deep learning-based automatic segmentation algorithm were 4434.24 mm^2^ (SD 746.07), 3676.43 mm^2^ (SD 914.68), and 9467.36 mm^2^ (SD 1962.98) for the same sequences, respectively.

**Figure 4:**
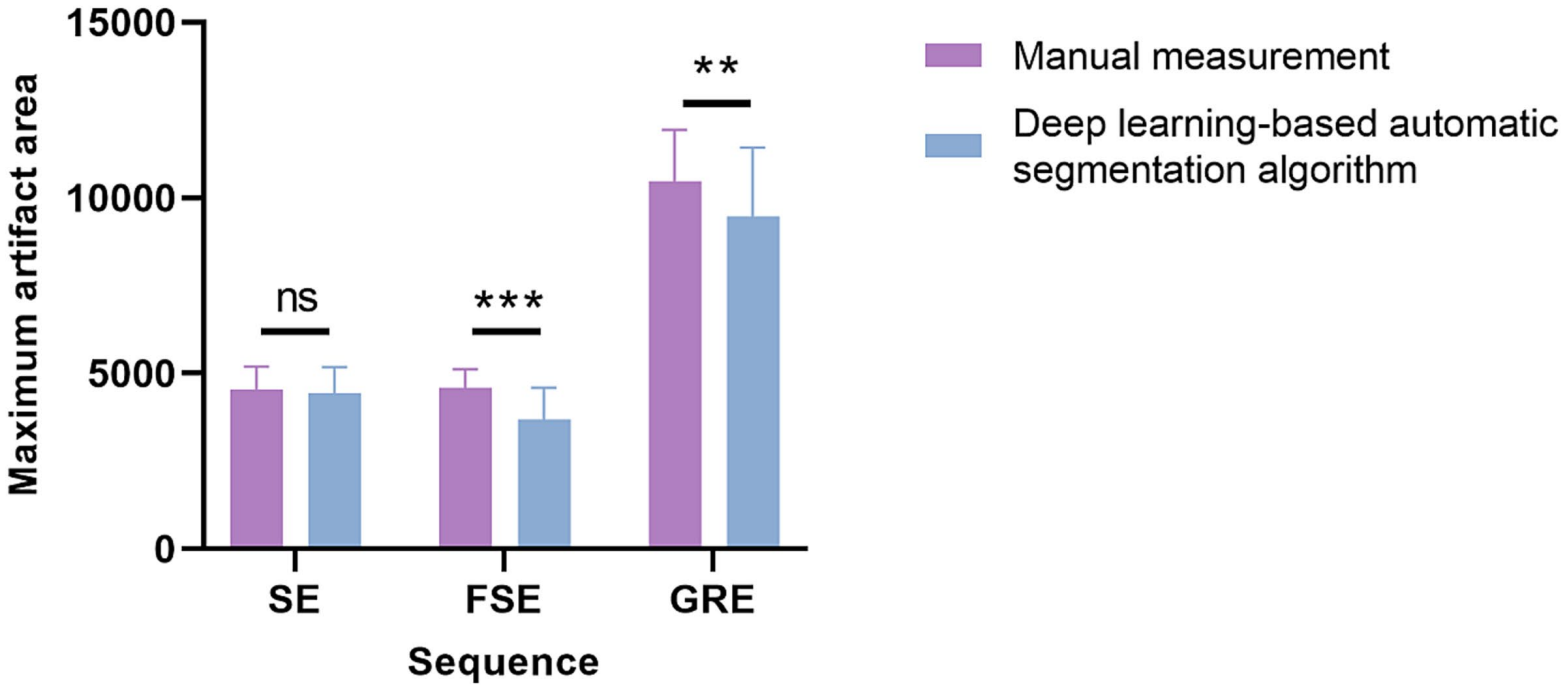
The maximum artifact area across MRI sequences for 12 subjects at 3.0 T. ***p < 0.001 **p < 0.01 “ns” means no significant difference.

Comparative analysis revealed no statistically significant difference between the manual and automated measurements for the SE sequences (95% CI [−151.21, 369.03], *p* = 0.377, Cohen’s d = 0.266). Significant discrepancies were observed for both the FSE sequences (95% CI [483.68, 1323.37], *p* < 0.001, Cohen’s d = 1.37) and GRE sequences (95% CI [397.36, 1595.80], *p* = 0.004, Cohen’s d = 1.057) (Figure 4).

### 3.3 Radiological evaluation of artifact impact on diagnostic utility

The inter-rater reliability was 0.812 (*p* < 0.001), suggesting a strong consistency between the two evaluators’ assessments.

Anatomical structure visibility was assessed by two experienced neuroradiologists independently using images acquired from 12 volunteers scanned with different MRI sequences at 1.5 T and 3.0 T. The resulting scores were statistically analyzed, with findings presented in Tables 3, 4. These results highlight the influence of CI artifacts on diagnostic image quality across different field strengths and MRI protocols.

**Table 3:** Visualization assessment of anatomical structures at 1.5 T MRI (mean ± SD).

Structure	Ipsilateral structure				Contralateral structure			
	SE	FSE	GRE	*p* value	SE	FSE	GRE	*p* value
Cerebellar hemisphere	A (1.17 ± 0.58) ^b^	A (1.5 ± 0.67) ^b^	NA (3 ± 0) ^b^	< 0.001***^c^	HQ (0 ± 0) ^b^	HQ (0 ± 0) ^b^	NA (3 ± 0) ^a^	< 0.001***^c^
Pons/medulla	HQ (0 ± 0) ^b^	HQ (0 ± 0) ^b^	NA (3 ± 0) ^b^	<0.001***^c^	HQ (0 ± 0) ^b^	HQ (0 ± 0) ^b^	NA (3 ± 0) ^b^	< 0.001***^c^
Midbrain	HQ (0.08 ± 0.29) ^b^	HQ (0 ± 0) ^b^	NA (3 ± 0) ^b^	< 0.001***^c^	HQ (0 ± 0) ^b^	HQ (0 ± 0) ^b^	NA (3 ± 0) ^b^	< 0.001***^c^
Deep gray matter (thalamus/basal ganglia)	HQ (0.58 ± 0.52) ^b^	HQ (0.67 ± 0.49) ^b^	NA (2.83 ± 0.39) ^b^	< 0.001***^c^	HQ (0 ± 0) ^b^	HQ (0 ± 0) ^b^	NA (1.67 ± 0.65) ^b^	< 0.001***^c^
Hippocampus	A (1.08 ± 0.52) ^a^	A (1.17 ± 0.58) ^b^	NA (3 ± 0) ^b^	< 0.001***^c^	HQ (0 ± 0) ^b^	HQ (0 ± 0) ^b^	NA (2.67 ± 0.49) ^a^	< 0.001***^c^
Frontal lobe	HQ (0.17 ± 0.39) ^b^	HQ (0.17 ± 0.39) ^b^	NA (2.09 ± 0.29) ^b^	< 0.001***^c^	HQ (0 ± 0) ^b^	HQ (0 ± 0) ^b^	A (1.17 ± 0.58) ^b^	< 0.001***^c^
Parietal lobe	NA (2 ± 0.43) ^b^	NA (2.08 ± 0.29) ^b^	NA (3 ± 0) ^b^	< 0.001***^c^	HQ (0 ± 0) ^b^	HQ (0 ± 0) ^b^	NA(2.08 ± 0.29) ^a^	< 0.001***^c^
Occipital lobe	NA (2.67 ± 0.49) ^b^	NA (2.67 ± 0.49) ^b^	NA (3 ± 0) ^b^	0.018*^d^	HQ (0 ± 0) ^b^	HQ (0 ± 0) ^b^	NA (3 ± 0) ^a^	< 0.001***^c^
Temporal lobe	NA (2 ± 0) ^b^	NA (2 ± 0) ^b^	NA(3 ± 0) ^b^	< 0.001***^c^	HQ (0 ± 0) ^b^	HQ (0 ± 0) ^b^	A (1.08 ± 0.52) ^a^	< 0.001***^c^
Optic nerve	HQ (0 ± 0) ^b^	HQ (0 ± 0) ^b^	NA (1.92 ± 0.9) ^a^	< 0.001***^c^	HQ (0 ± 0) ^b^	HQ (0 ± 0) ^b^	A (0.92 ± 1.08) ^a^	< 0.001***^d^
Internal auditory canal	HQ (0.08 ± 0.29) ^b^	HQ(0.08 ± 0.29) ^b^	NA (3 ± 0) ^b^	< 0.001***^c^	HQ (0 ± 0) ^b^	HQ (0 ± 0) ^b^	NA (2.83 ± 0.39) ^a^	< 0.001***^c^
Corpus callosum	HQ (0 ± 0) ^b^ (Ant)	HQ (0 ± 0) ^b^ (Ant)	A (1.42 ± 0.79) ^b^ (Ant)	< 0.001***^c^	A(0.92 ± 0.29) ^a^ (Pos)	A (1 ± 0) ^b^ (Pos)	NA (3 ± 0) ^b^ (Pos)	< 0.001***^c^

*p < 0.05; ***p < 0.001; ^a^*p* < 0.05 when compared with 3.0 T group; ^b^*p* > 0.05 when compared with 3.0 T group; ^c^indicates significant differences in Friedman test with post-hoc results showing GRE > SE and GRE > FSE; ^d^represents significant Friedman test results without significant post-hoc differences, but Wilcoxon signed-rank test confirmed GRE > SE and GRE > FSE; Ant denotes anterior corpus callosum; Pos indicates posterior corpus callosum; Mean represents mean value; SD stands for standard deviation; HQ refers to high-quality images with mean scores ≤ 0.75; A indicates images with artifacts (0.75 < mean score ≤ 1.5) that remain diagnostically acceptable; NA represents non-assessable images (1.5 < mean score ≤ 3).

**Table 4:** Visualization assessment of anatomical structures at 3.0 T MRI (mean ± SD).

Structure	Ipsilateral structure				Contralateral structure			
	SE	FSE	GRE	*p* value	SE	FSE	GRE	*p* value
Cerebellar hemisphere	A (1.33 ± 0.89)	A (1.33 ± 0.99)	NA (2.92 ± 0.29)	< 0.001***^c^	HQ (0 ± 0)	HQ (0 ± 0)	A (1.5 ± 0.91)	< 0.001***^c^
Pons/medulla	HQ (0 ± 0)	HQ (0 ± 0)	NA (2.75 ± 0.45)	< 0.001***^c^	HQ (0 ± 0)	HQ (0 ± 0)	NA (2.33 ± 1.16)	< 0.001***^c^
Midbrain	HQ (0 ± 0)	HQ (0 ± 0)	NA (3 ± 0)	<0.001 ***^c^	HQ (0 ± 0)	HQ (0 ± 0)	NA (2.83 ± 0.39)	< 0.001***^c^
Deep gray matter (thalamus /basal ganglia)	A (0.83 ± 0.39)	HQ (0.75 ± 0.45)	NA (2.75 ± 0.45)	< 0.001***^c^	HQ (0 ± 0)	HQ (0 ± 0)	A (1.5 ± 0.52)	< 0.001***^c^
Hippocampus	HQ (0.58 ± 0.52)	A (1.33 ± 0.78)	NA (3 ± 0)	< 0.001***^c^	HQ (0 ± 0)	HQ (0 ± 0)	A (1.42 ± 1.084)	< 0.001***^c^
Frontal lobe	HQ (0.67 ± 0.65)	HQ (0.75 ± 0.75)	NA (2 ± 0.74)	< 0.001***^c^	HQ (0 ± 0)	HQ (0 ± 0)	HQ (0.67 ± 0.78)	0.002*^d^
Parietal lobe	NA (2.42 ± 0.79)	NA (2.42 ± 0.79)	NA (3 ± 0)	0.016*^d^	HQ (0 ± 0)	HQ (0 ± 0)	A (1.08 ± 0.29)	< 0.001***^c^
Occipital lobe	NA (2.33 ± 1.16)	NA (2.33 ± 1.16)	NA (2.92 ± 0.29)	0.018*^e^	HQ (0 ± 0)	HQ (0 ± 0)	NA (2 ± 0.95)	< 0.001***^c^
Temporal lobe	NA (1.92 ± 0.29)	NA (1.75 ± 0.45)	NA (2.92 ± 0.29)	< 0.001***^c^	HQ (0 ± 0)	HQ (0 ± 0)	HQ (0.42 ± 0.52)	0.007*^d^
Optic nerve	HQ (0 ± 0)	HQ (0 ± 0)	A (0.83 ± 0.84)	< 0.001***^d^	HQ (0 ± 0)	HQ (0 ± 0)	HQ (0 ± 0)	
Internal auditory canal	HQ (0 ± 0)	HQ (0.08 ± 0.29)	NA (3 ± 0)	< 0.001***^c^	HQ (0 ± 0)	HQ (0 ± 0)	HQ (0.42 ± 0.67)	0.018*^e^
Corpus callosum	HQ (0 ± 0) (Ant)	HQ (0 ± 0) (Ant)	A (1.17 ± 1.34) (Ant)	0.002*^d^	HQ (0.58 ± 0.52) (Pos)	HQ (0.58 ± 0.67) (Pos)	NA (3 ± 0) (Pos)	< 0.001***^c^

*p < 0.05 and ***p < 0.001 indicate significance levels; ^c^denotes significant Friedman test results with post-hoc analysis showing GRE > SE and GRE > FSE; ^d^represents significant Friedman test results without significant post-hoc differences, though Wilcoxon signed-rank test confirmed GRE > SE and GRE > FSE; ^e^indicates significant Friedman test results without significant post-hoc differences, and Wilcoxon signed-rank test showed no significant differences among SE, FSE and GRE sequences (*p* > 0.05). Ant denotes anterior corpus callosum; Pos indicates posterior corpus callosum; Mean represents mean value; SD stands for standard deviation; HQ refers to high-quality images with mean scores ≤ 0.75; A indicates images with artifacts (0.75 < mean score ≤ 1.5) that remain diagnostically acceptable; NA represents non-assessable images (1.5 < mean score ≤ 3).

At 1.5 T MRI, all contralateral anatomical structure scores for the images acquired with SE and FSE sequences were ≤ 0.75 (mean), except for the posterior corpus callosum. For the GRE sequences, the contralateral scores were 1.5–3 (mean), except in the frontal lobe, temporal lobe, and optic nerve. At 1.5 T, the ipsilateral anatomical structure scores for the SE and FSE sequences were 0–1.5 (mean), except in the parietal lobe, occipital lobe, and temporal lobe. For the GRE sequences, all ipsilateral scores were 1.5–3 (mean), except for the anterior corpus callosum (Table 3).

At 1.5 T MRI, significant intergroup differences were detected among all of the anatomical structures in the images acquired with SE, FSE, and GRE sequences (*p* < 0.05). Post-hoc comparisons revealed that the GRE sequences yielded significantly higher scores compared to both the SE and FSE sequences (*p* < 0.05), whereas no significant difference was noted between the SE and FSE sequences (*p* > 0.05) (Table 3). For post-hoc Wilcoxon signed-rank tests at 1.5 T, effect sizes (r) were large for all comparisons involving GRE sequences (r = 0.66–0.88), while comparisons between SE and FSE sequences showed small to medium effects (r = 0.00–0.53).

At 3.0 T MRI, both the SE and FSE sequences demonstrated a mean score of ≤ 0.75 for all contralateral anatomical structures. For the GRE sequences, contralateral structures exhibited mean scores of 0 ≤ mean ≤ 1.5, except for the pons/medulla, midbrain, occipital lobe, and posterior corpus callosum. Regarding the ipsilateral structures at 3.0 T, the SE and FSE sequences yielded a mean score of 0 ≤ mean ≤ 1.5 for all regions, except for the parietal, occipital, and temporal lobes. The GRE sequence produced higher scores (1.5 < mean ≤ 3) for all of the ipsilateral structures, except the optic nerve and anterior corpus callosum (Table 4).

At 3.0 T MRI, statistically significant intergroup differences (*p* < 0.05) were observed among the SE, FSE, and GRE sequences for all anatomical structures, except for the contralateral optic nerve, which exhibited no significant difference (all image quality scores = 0). Post-hoc comparisons demonstrated that the GRE sequences yielded significantly higher image quality scores than both SE and FSE sequences (*p* < 0.05) for all the structures, except the ipsilateral occipital lobe with CI and the contralateral internal auditory canal. No significant differences were noted between the SE and FSE sequences for any anatomical structures (*p* > 0.05) (Table 4). For post-hoc Wilcoxon signed-rank tests at 3.0 T, effect sizes for comparisons involving GRE sequences were also large for most structures (r = 0.51–0.88, with the majority exceeding 0.80), while comparisons between SE and FSE sequences showed small to medium effects (r = 0.00–0.46).

Except for specific structures (such as the ipsilateral hippocampus in the SE sequence, optic nerve in the GRE sequence, and contralateral cerebellar hemisphere, hippocampus, parietal lobe, occipital lobe, temporal lobe, internal auditory canal, optic nerve in the GRE sequence, along with posterior corpus callosum in the SE sequence under CI conditions) exhibited significant differences in the image quality (*p* < 0.05) between 1.5 T and 3.0 T MRI across the sequences, and the remaining structures demonstrated no statistically significant differences in the image quality data (*p* > 0.05) between 1.5 T and 3.0 T MRI scans (Table 3).

## 4 Discussion

Cochlear implantation has become the standard surgical procedure for rehabilitation of patients with severe to profound sensorineural hearing loss. Nevertheless, MRI scans at 1.5 T or 3.0 T following CI surgery can produce artifacts, making it challenging to assess the anatomical structures of large brain areas (22).

This research estimated the maximum dimension and area of artifacts to clinically determine whether scanning protocols require adjustment and indirectly assess implant displacement, providing timely warnings of post-CI implantation complications. Significant differences were observed in artifact size among various MRI sequences, which are consistent with the findings reported in the cadaver study by Majdani et al. (14, 16, 17). GRE sequences produced significantly larger artifacts than SE and FSE sequences; in contrast, the difference between SE and FSE sequences was minimal. The GRE sequence is mainly used clinically for detecting intracranial hemorrhage, iron deposition, and calcifications, and it is also widely applied in functional MRI (fMRI) and magnetic resonance angiography (MRA). However, its high sensitivity to magnetic susceptibility effects may account for the more pronounced artifacts observed in GRE compared with other sequences.

The comparable artifact dimensions between 1.5 T and 3.0 T suggest a nonlinear relationship between field strength and susceptibility artifact size. Gottfried et al. similarly reported that CI-induced artifact dimensions do not increase uniformly with field strength, implying a possible plateau effect beyond 1.5 T (23). This may be explained by limitations in spatial resolution, partial volume effects, and field strength-dependent changes in artifact morphology, which prevent linear translation of frequency shifts into artifact size. Additionally, for implants containing permanent magnets such as the CS-30A, the magnet itself is the primary source of artifacts, and its magnetization may already approach saturation at 1.5 T, limiting further artifact increase at 3.0 T. In this investigation, the maximum artifacts produced by the CS-30A CI in the axial plane under 1.5 T MRI scanning (SE and FSE sequences) were numerically different from the findings of Sharon et al. (24), who reported anteroposterior and mediolateral dimensions of 4.6 cm and 3.6 cm, respectively, using a different CI model and scanner settings. However, the measurements agreed with those reported by Majdani et al. (15) in axial plane T1-weighted and T2-weighted sequence scans. The comparison of artifacts across different MRI field strengths and sequences is challenging for several reasons: (1) only a few studies have determined the artifact size. (2) Descriptions of measurement procedures are often brief and vary across studies. For example, Canzi et al. (25) measured artifacts by fitting a circle to the edge of the signal void in axial T1w and T2w TSE sequences and computing the maximum radius. (3) Different studies employed distinct CI models.

This work is the first to utilize a deep learning–based automatic segmentation algorithm for calculating and evaluating artifact area. At a field strength of 3.0 T, the artifact area in SE sequences showed high consistency between manual measurements and the deep learning–based automatic segmentation algorithm, affirming the reliability of algorithm-assisted assessment and providing a feasible technical method for future large-scale clinical studies. Compared with manual measurements, the deep learning–based automatic segmentation algorithm offers advantages in time efficiency, as it can process a large number of images more rapidly while maintaining excellent agreement with manual annotation. However, this algorithm relies on image quality, and factors such as image distortion, low resolution, and noise can interfere with its performance. Therefore, further large-sample measurement and learning are required for optimization.

The image quality of most contralateral anatomical structures was higher than that of ipsilateral structures across both field strengths (SE and FSE sequences). This finding suggests that CI artifacts have a substantially greater impact on ipsilateral anatomical regions than on contralateral ones. In addition, the anterior corpus callosum was less affected by CI artifacts than the posterior portion, which is aligned with Dewey et al.’s (19) finding that “the anterior corpus callosum remains relatively unaffected by CI regardless of position.” This observation highlights the importance of paying special attention to imaging quality in the tissues surrounding the implant side during radiological assessment. Our experimental procedure (external CI fixation using a swim cap) was comparable to that of Dewey et al. (19). Nevertheless, they focused on the effect of implant position on artifact size using a different CI model (Cochlear Nucleus) and a cadaveric head model at 3.0 T, whereas we systematically compared artifact dimensions across three sequences and two field strengths in healthy volunteers using the Nurotron CS-30A implant. Under both 1.5 T and 3.0 T MRI (SE and FSE sequences), the parietal, occipital, and temporal lobes on the CI-ipsilateral side exhibited the lowest image visibility. Other structures, such as the internal auditory canal and optic nerves on the ipsilateral side, were partially obscured by the artifacts; however, their image quality was considered acceptable. These findings agree to a certain extent with previous studies on various CI models (25, 26).

Of note, the 9 cm placement distance used in this study is consistent with the finding by Yun et al. that this distance and beyond limits the impact of the artifact on visualizing the inner ear (27), further supporting that our experimental setup reflects clinically relevant conditions.

Comprehending the extent of CI-induced artifacts in these commonly used clinical sequences can aid in optimizing scanning protocols in CI recipients who may require future imaging. Most structures did not show significant differences in image quality scores between 1.5 T and 3.0 T MRI. This finding agrees only partially with those from previous studies (28), possibly due to differences in sequence parameters, CI models, and scanner platforms across studies.

These insights may aid in selecting the most suitable MRI field strengths and sequences for CI recipients. For instance, when acquiring axial MRI images, the GRE sequence should be avoided for CI recipients (13). Instead, the SE or FSE sequence should be prioritized to minimize artifact interference with anatomical visibility. Selecting metal artifact reduction sequences, such as MARS, MAVRIC-SL, and SEMAC (29), which have been proven to significantly reduce artifacts, could be beneficial. For the Nurotron CS-30A implant, the magnet can be removed by a qualified surgeon if the artifact substantially compromises diagnostic quality, similar to the removable magnet designs available from other CI manufacturers (12, 13). However, if GRE sequences must be used for specific clinical indications (e.g., detection of hemorrhage or calcification), optimizing acquisition parameters such as shortening TE, increasing the frequency matrix, and reducing slice thickness may help mitigate susceptibility artifacts (20), though image quality may remain suboptimal in CI recipients. Optimization of MRI sequences for CI recipients can significantly improve the visibility of adjacent brain structures, thereby enhancing diagnostic accuracy for neurological disorders such as infection, tumor, and ischemia. The reduction of artifact interference also facilitates postoperative evaluation and long-term follow-up, supporting clinical decision-making and patient management. Furthermore, a deeper understanding of artifact characteristics under different imaging conditions may inform MRI safety guidelines, sequence selection, and implant design, ultimately promoting safer and more efficient imaging evaluation for cochlear implant patients.

### 4.1 Limitation

This study has several limitations. First, the cochlear implant was externally fixed onto the scalp rather than surgically implanted into the temporal bone. This ex-vivo simulation model does not fully replicate postoperative conditions, where the implant is in direct contact with bone and covered by soft tissue. The external placement may alter the distance between the implant magnet and the brain parenchyma, potentially affecting the extent and pattern of MRI artifacts, and thus may change the degree of obscuration of the examined anatomical structures. Caution is warranted when generalizing these findings to clinical CI recipients. Future studies should validate these results in implanted patients. Second, this study included only axial MRI acquisitions using planar SE, FSE, and GRE sequences, without evaluating 3D sequences or other clinically relevant sequences such as FLAIR. Since 3D sequences provide higher spatial resolution and allow multiplanar reconstruction, and FLAIR is clinically important for detecting white matter lesions, inflammation, and meningeal abnormalities, the absence of these sequences may limit the generalizability of the findings to broader clinical applications. Our future studies will incorporate a wider range of three-dimensional and clinically used sequences to comprehensively assess the impact of different imaging parameters and sequences on MRI artifacts caused by cochlear implants, thereby providing a more robust basis for optimizing clinical imaging protocols. Third, the use of different scanners (SIEMENS at 1.5 T vs. GE at 3.0 T) and different slice thicknesses (6 mm vs. 3 mm) may confound direct comparisons between the two field strengths. Future studies using the same scanner platform with identical parameters at both field strengths are needed to confirm the present findings. Moreover, the relatively small sample size (*n* = 12) may explain the lack of statistically significant differences in image quality scores for the ipsilateral occipital lobe and contralateral internal auditory canal among SE, FSE, and GRE sequences under 3.0 T cranial MRI. And the relatively small sample size may reduce the statistical power of the study, making it difficult to detect subtle differences between groups. Although the sample size of this study is relatively limited, considering the increasing number of patients receiving the MR Conditional cochlear implant CS-30A after its market release, the results of this study still hold important practical significance and provide experimental evidence for the safety and reliability of MRI examinations in cochlear implant recipients. Additionally, our analysis focused on signal loss artifacts, whereas image distortion (e.g., periodic shadowing) was not systematically evaluated. Distorted areas may still retain limited diagnostic value (14), and future studies should consider both signal loss and distortion when assessing artifact impact.

### 4.2 Conclusion

This study quantified the maximum dimension of artifacts and, for the first time, utilized a deep learning–based automatic segmentation algorithm to calculate and evaluate the areas of artifacts. The artifact areas in SE sequences exhibited high consistency between manual measurements and the deep learning–based automatic segmentation algorithm. Under both 1.5 T and 3.0 T MR scans, the CI produced larger artifact areas in GRE sequences compared with SE and FSE sequences, considerably reducing the visibility of adjacent anatomical structures, with the ipsilateral parietal, occipital, and temporal lobes exhibiting the lowest visibility. The 1.5 T and 3.0 T field strengths had little impact on the distribution of CI artifacts. Ipsilateral anatomical structures were more affected by artifacts than the contralateral side. Therefore, when selecting an MR scanning protocol for CI recipients, SE and FSE sequences should be prioritized over GRE sequences. Both 1.5 T and 3.0 T field strengths are feasible, as artifact sizes were comparable between them. The choice of field strength should be guided by clinical needs and scanner availability, with 3.0 T offering potential SNR advantages for specific diagnostic tasks when appropriate sequences are used.

## Data Availability

The original contributions presented in the study are included in the article/supplementary material, further inquiries can be directed to the corresponding authors.
